# Effect of Hepatitis E Virus RNA Universal Blood Donor Screening, Catalonia, Spain, 2017‒2020

**DOI:** 10.3201/eid2801.211466

**Published:** 2022-01

**Authors:** Marta Bes, Maria I. Costafreda, Mar Riveiro-Barciela, Maria Piron, Angie Rico, Josep Quer, Lluis Puig, Silvia Sauleda

**Affiliations:** Instituto de Salud Carlos III, Madrid, Spain (M. Bes, M.I. Costafreda, M. Riveiro-Barciela, M. Piron, A. Rico, J. Quer, L. Puig, S. Sauleda);; Vall d’Hebron Barcelona Hospital Campus, Barcelona, Spain (M. Bes, M.I. Costafreda, M. Riveiro-Barciela, M. Piron, A. Rico, J. Quer, L. Puig, S. Sauleda);; Banc de Sang i Teixits, Catalonia, Spain (M. Bes, M.I. Costafreda, M. Piron, A. Rico, L. Puig, S. Sauleda)

**Keywords:** hepatitis E virus, HEV, viruses, virus RNA, viral load, universal blood donor screening, blood donors, screening, IgG, IgM, acquisition route, Catalonia, Spain

## Abstract

Hepatitis E virus (HEV) is the major cause of acute viral hepatitis in several countries in Europe. HEV is acquired mainly by consumption of contaminated pork but can also be transmitted through blood transfusion. HEV infection is usually self-limited but can become persistent in immunocompromised persons. During the first 30 months of HEV RNA universal screening of blood donations in Catalonia, Spain, we identified 151 HEV RNA–positive donations (1/4,341 blood donations). Most infected donors reported consumption of pates and sausages, and 58% were negative for HEV IgM and IgG. All HEV isolates belonged to genotype 3. All infected donors spontaneously resolved the infection, and no neurologic symptoms and reinfections were observed after 1 year of follow-up. Since the implementation of HEV RNA universal screening, no new cases of transfusion-transmitted HEV infection were reported. Our data indicate HEV screening of blood donations provides safer blood for all recipients, especially for immunosuppressed persons.

Hepatitis E virus (HEV; family Hepeviridae, genus *Orthohepevirus*) (*1*) is an RNA virus that causes acute viral hepatitis in humans. Five HEV genotypes are known to infect humans. Genotypes 1 and 2 are transmitted by the fecal‒oral route and cause large waterborne outbreaks in developing countries. Genotype 3 and 4 cause zoonotic infections transmitted from infected animals, such as pigs, deers, and wild boars, to humans. Transmission usually occurs through consumption of raw or inadequately cooked processed pork meat (*2*,*3*). HEV genotype 7 has been identified mainly in camels but also an immunocompromised transplant patient (*4*). Several cases of transfusion-transmitted HEV infections have been reported; these infections might cause chronic hepatitis in immunosuppressed recipients (*5*) and were not prevented by pathogen-reduction methods (*6*).

In Europe, most acute HEV infections are caused by genotype 3 (*7*). Although these infections are usually asymptomatic in immunocompetent persons, HEV infection poses a particular risk to persons who have compromised immune systems because they might have persistent infection develop, with rapid progression to cirrhosis, decompensation, and death (*8*). Moreover, HEV genotype 3 infection has been related to several extrahepatic manifestations, such as Guillain-Barré syndrome, inflammatory polyradiculopathy, bilateral brachial neuritis, encephalitis, ataxia/proximal myopathy, and necrotizing myositis (*8*). There is no proven treatment for chronic HEV infection, although ribavirin therapy or reduction of immunosuppression have been successful in achieving HEV RNA clearance (*9*).

A major systematic study assessing HEV transmission through blood transfusion was conducted in England during 2012–2013 (*10*). The study reported that 42% of recipients who received HEV RNA‒contaminated blood components showed evidence of infection, and progression to a chronic infection was demonstrated in 50% of HEV-infected immunosuppressed recipients. The study also observed a direct correlation between HEV viral load and plasma transfused volume (HEV RNA infusion dose), and the capability to induce transfusion-transmitted HEV infection (*10*).

Moreover, a tertiary hospital in Catalonia, Spain, reported 2 cases in immunocompetent patients who had acute hepatitis E resulting from HEV transmitted by erythrocyte concentrate or cryosupernatant plasma (*11**,**12*). The HEV infection resolved spontaneously (*11*) or after ribavirin therapy (*12*).

HEV RNA screening of blood donations has been in place for the last few years in the Netherlands, some blood banks in Germany, Ireland, the United Kingdom, France, Catalonia, and Japan (*13*). However, there is no international consensus on sensitivity required for HEV RNA. Therefore, blood banks have chosen to implement either individual or pool testing. Furthermore, HEV RNA screening is exceptional with respect to other screening markers in blood donations, for which a positive result (i.e., confirmed HIV antibodies, hepatitis B virus surface antigen, or hepatitis C virus antibodies) permanently defers the donor. HEV RNA‒positive blood donors are deferred for 6 months in Catalonia.

We report the results of routine screening for HEV RNA in Catalonia during November 2017‒April 2020. We aimed to determine the prevalence of HEV among blood donors in Catalonia and to describe epidemiology and progression of HEV acute infection in immunocompetent and asymptomatic blood donors.

## Methods

### Screening and Characterization of Donor Samples

During November 1, 2017‒April 30, 2020, we tested 655,523 blood donations, plasma samples, and platelet apheresis samples in Catalonia for HEV RNA by using the Procleix HEV assay (Grifols Diagnostic Solutions Inc., https://www.diagnostic.grifols.com/en/home) in pools of 16 samples (95% of limit of detection 176 IU/mL in individual donor when tested in pools of 16 samples). We analyzed individually all samples in reactive pools by using the same assay (95% of limit detection 11 IU/mL in individual samples) and selected HEV RNA‒containing blood components.

We assessed HEV RNA confirmation and viral load by using an in-house quantitative reverse transcription PCR (RT-PCR) for the open reading frame 3 region adapted from Slot et al. (*14*). The lower limit of detection (95% cutoff value) was 7.5 IU/mL for a 9.6-mL plasma volume and 45 IU/mL for a 1.6-mL plasma volume. We calculated HEV viral loads by using a calibration curve based on the first World Health Organization International Standard for HEV RNA (PEI code 6329/10; https://www.pei.de). We performed HEV serologic testing by using Mikrogen IgM and IgG Detection Assays (https://www.mikrogen.de) and biochemical analysis (levels of direct bilirubin, total bilirubin, aspartate aminotransferase, alanine aminotransferase, and γ-glutamyltransferase) for all HEV RNA‒positive donations.

### Archived Samples

Some donors might have had a previous (archived) blood donation within 6 months before HEV RNA identification. We retested these archived plasma samples by using the HEV RNA Procleix HEV assay for individual samples.

### Follow-up of HEV‒Infected Donors

We collected age, sex, postal code, and country of birth for all HEV RNA‒positive donors. All donors received a notification to follow-up on their HEV infection at the blood bank in 1 month. HEV RNA tested individually, HEV IgG and IgM, and biochemical parameters were reanalyzed for the follow-up sample. At the time of follow-up, donors completed a questionnaire evaluating symptoms of acute hepatitis, meat consumption, type of meat products (sausages, pates), type of dwelling (apartment, house, and farm), wastewater system (mains sewer, septic tank), and travel history outside Catalonia 2 months before blood donation.

In addition, donors who were still HEV RNA reactive at follow-up received the opportunity to visit a clinician at the liver unit of Vall d’Hebron Hospital between 2 and 6 months after HEV RNA detection in the blood bank. The clinician evaluated the hepatic and extrahepatic symptoms related to acute HEV infection, and another serum sample was obtained to determine the presence of HEV RNA, HEV IgG and IgM, and the biochemical parameters. If otherwise qualified, donors were reinstated for donation 6 months after the last HEV RNA‒positive result.

### Genotyping

We amplified a major fragment of reading frame 2 (nt positions 5145‒7127) by using a nested reverse transcription nested RT-PCR. We performed reverse transcription and the first PCR by using the SuperScript III One-Step RT-PCR System with Platinum Taq DNA Polymerase (Invitrogen, https://www.thermofisher.com) and 0.35 nmol/L of each primer (forward: 5′-CCGACAGAATTGATTTCGTCGGC-3′ and reverse: 5′-ACTCCCGRGTYTTACCYACCTT-3′). We performed a nested PCR by using Platinum Taq DNA Polymerase (Invitrogen), 0.32 nmol/L of each primer (forward: 5′-TCGTCTCAGCCAATGGCGAGCC-3′ and reverse: 5′-CASARAANGTCTTNGARTACTGCT-3′), and 30 cycles of standard PCR conditions. We loaded amplicons onto a 2% agarose gel, subjected the amplicons to electrophoresis, and purified specific bands by using the QIAquick Gel Extraction Kit (QIAGEN, https://www.qiagen.com). We sequenced purified material by using the Sanger method and a 3130xL Genetic Analyzer System (Applied Biosystems, https://www.thermofisher.com).

### Ethics

We obtained signed consent for blood donation from each donor at the time of donation. Donors who accepted the follow-up at the Liver Unit of Vall d’Hebron Hospital (Barcelona, Spain) signed an additional research informed consent. The Clinical Research Institutional Review Board of Vall d’Hebron University Hospital in Barcelona approved the study (PR[BST]351/2017).

### Statistical Analysis

Prevalence was expressed as percentages and 95% CIs. Quantitative variables were expressed as means, medians, and SDs. We compared percentages by using the χ^2^ test. p values <0.05 were considered significant.

## Results

### Prevalence of HEV Acute Infections in Blood Donors

**Figure 1:**
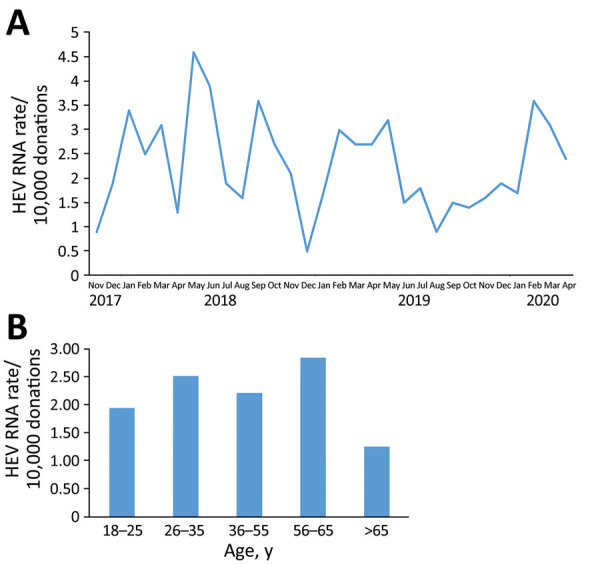
Rate of HEV RNA‒positive donations, Catalonia, Spain, November 2017‒April 2020. A) All donations; B) by age group during the same period. HEV, hepatitis E virus.

During November 1, 2017‒April 30, 2020, we screened 655,523 blood donations HEV RNA in pools of 16 samples. All reactive pools were resolved to an individual reactive donation, which was confirmed by quantitative RT-PCR. During the 30-month period, we selected 151 HEV RNA‒positive donations representing an overall prevalence of 1 case/4,341 donations (95% CI 1 case/3,703 donations‒1 case/5,004 donations). The prevalence of HEV RNA‒positive donations according to month of detection during the 2.5 years of the study fluctuated, and no seasonality of HEV cases was observed (Figure 1, panel A).

### Demographic Data for HEV RNA‒Positive Donors

Most (62.9%) donors with acute HEV infection were men; mean age ± SD was 41.5 ± 12.9 years, and 92.7% were born in Spain (Table 1). A significantly higher proportion of men were infected than women (95/346,592 vs. 56/308,931; p = 0.0168). Although HEV RNA was detected in all donor age groups, the highest rate was observed in persons 56–65 years of age (Figure 1, panel B).

**Table 1:** Characteristics for 151 HEV RNA‒positive blood donors, Catalonia, Spain*

Characteristic	Value
Sex	
** M**	95 (62.9)
** F**	56 (37.1)
Mean ± SD age, y	41.5 ± 12.9
Country of birth	
** Spain**	140 (92.7)
** Argentina**	4 (2.6)
** Uruguay **	2 (1.3)
** Romania**	2 (1.3)
** Germany**	1 (0.7)
** Portugal**	1 (0.7)
** France**	1 (0.7)
Median viral load, IU/mL (range)	503 (40–999,340)
HEV IgM/IgG	
** HEV IgM positive**	63 (42.0)
** HEV IgG positive**	45 (30.0)
Biochemical parameters	
** Bilirubin >1.1 mg/dL**	4 (2.6)
** AST >80 U/L**	10 (6.6)
** ALT >80 U/L**	15 (9.9)
** γ-glutamyltransferase > 55 U/L **	24 (15.9)

*Values are no. (%) unless indicated otherwise. ALT, alanine aminotransferase; AST, aspartate aminotransferase; HEV, hepatitis E virus.

**Figure 2:**
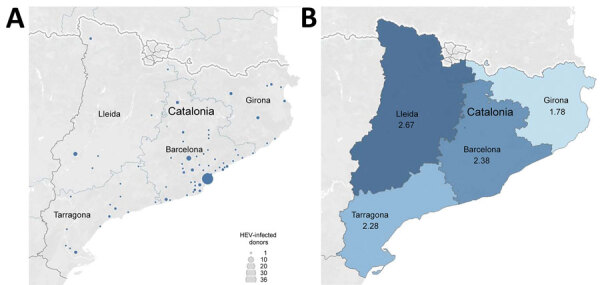
Geographic distribution of HEV-infected blood donors, Catalonia, Spain, November 2017‒April 2020. A) All donations (n = 151). B) HEV RNA detection rate (per 10,000 analyzed blood donations), by province (Barcelona, Girona, Lleida, and Tarragona). Maps were created by using Tableau Software (https://www.tableau.com). HEV, hepatitis E virus.

HEV-infected blood donors were evenly distributed by province: 107 donors in Barcelona, 18 in Tarragona, 14 in Girona, and 12 in Lleida (Figure 2, panel A). The prevalence of HEV RNA per 10,000 donations varied from 1.78 in Girona to 2.67 in Lleida (Figure 2, panel B).

### Virologic Parameters for HEV RNA‒Positive Donors

**Figure 3:**
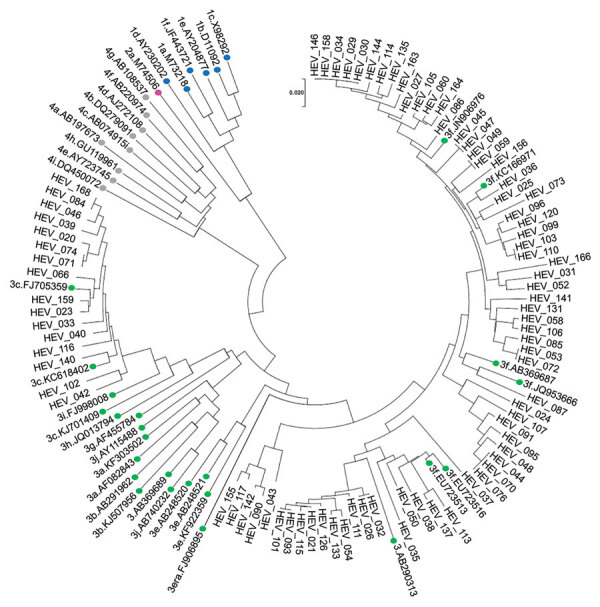
Phylogenetic analysis of HEV isolates from HEV-positive blood donors identified, Catalonia, Spain, November 2017‒April 2020 (n = 91). Evolutionary relationships of taxa. Evolutionary history was inferred by using the neighbor-joining method. Sequence analysis was performed by using MEGA 7 software (https://www.megasoftware.net) and HEV reference sequences described in Smith et al. (*15*), and additional HEV sequences from GenBank (labeled by accession number). Evolutionary distances were computed by using the maximum composite likelihood method, and the phylogenetic tree was constructed by using the neighbor-joining method. Colored dots indicate the HEV reference sequences used in the analysis (blue, HEV genotype 1; purple, HEV genotype 2; green, HEV genotype 3; gray, HEV genotype 4). Scale bar indicates nucleotide substitutions per site. HEV, hepatitis E virus.

The median viral load was 503 IU/mL (range 40–999,340 IU/mL) (Table 1). All HEV isolates that were successfully genotyped (91, 60.9%) belonged to genotype 3. Phylogenetic analysis showed that most (73, 80.2%) isolates clustered with HEV subgenotype 3f, whereas 18 (19.8%) isolates clustered with subgenotype 3c (Figure 3). Two isolates were 100% homologous and obtained from members of the same family who were probably infected simultaneously and from the same source inoculum.

### Serologic and Biochemical Parameters for HEV RNA‒Positive Donors

At the time of donation, 63 (42%) of HEV RNA–positive donors were positive for HEV IgM and 45 (30%) were positive for HEV IgG/IgM (Table 1). Increased levels of bilirubin were found in 4 (2.6%) of these HEV-infected donors, increased levels of alanine aminotransferase in 10 (6.6%), and increased levels of aspartate aminotransferase (AST) in 15 (9.9%). Twenty-four donors also had increased levels of γ-glutamyltransferase at the time of donation (Table 1).

### Clinical Symptoms and Donor Risk Factors for HEV Infection

A total of 111 (73.5%) HEV RNA‒positive donors completed the questionnaire with information about clinical symptoms, diet, type of housing, and travel history to determine possible routes of HEV acquisition. Only 2 donors reported symptoms of acute hepatitis, such as pain/aches or abdominal pain. However, 24.3% of HEV RNA donors reported general unrest and 15.3% reported dark urine or light stools during 1 or 2 weeks before or after donation (Table 2).

**Table 2:** Clinical characteristics and risk factors for HEV infection reported by HEV RNA‒positive donors, Catalonia, Spain*

Characteristic	No. (%) responses, n = 111
Symptoms or clinical signs	
** Symptom of acute hepatitis: aches, nausea, abdominal pain, vomiting**	2 (1.8)
** General unrest**	27 (24.3)
** Dark urine or light stool**	17 (15.3)
Information on diet: eating habits related with HEV acquisition	
** Vegetarian or vegan**	1 (0.9)
** Usual consumption of undercooked meat**	47 (42.3)

** Consumption of liver pate or sausages in last 2 months†**	63 (65.6)
** Consumption of seafood in last 2 months**	70 (63.0)

** Consumption of nonbottled water†**	51 (53.1)
** Consumption of bushmeat**	2 (1.8)
Visited a farm within 2 months before donation	11 (9.9)
Lived in rural areas or on farms	20 (18)
Traveled outside Spain within 2 months before donation	26 (23.4)

*HEV, hepatitis E virus. †96 HEV RNA‒positive donors responded to these questions.

One HEV RNA‒positive donor was vegetarian, and no risk factor could be associated with HEV infection. However, most HEV-infected donors consumed undercooked meat, especially pork (42.3%); liver pate or sausages (65.6%); or shellfish, especially mollusks (63%), 2 months before blood donation. Moreover, 50% of them reported consuming nonbottled water. In addition, 2 HEV RNA‒positive donors consumed game meat 1 month before blood donation.

Regarding travel history, 26 HEV-infected donors traveled outside Spain in the 2-month period before blood donation. Moreover, 11 persons visited farms 2 months before blood donation, and 20 persons lived on a farm or in a rural area.

### HEV RNA‒Positive Blood Donor Follow-up

During the first month after RNA detection, 127 HEV RNA‒positive donors attended follow-up appointments at a blood bank (Table 3). At this time, HEV IgM was found in 75% and IgG in 78% of retested donors; 37% had undetectable HEV RNA. All HEV RNA‒positive donors with altered levels of transaminases or bilirubin at the time of donation had levels that returned to standard levels at 1-month follow-up, while 16 HEV RNA‒positive donors with who had standard bilirubin or transaminase levels at the time of donation had increased bilirubin or transaminase levels at 1-month follow-up.

**Table 3:** Follow-up of HEV RNA positive blood donors, Catalonia, Spain*

Characteristic	Baseline	1-month follow-up	2-month follow-up	6-month follow-up	>1 year follow-up
No. persons	151	127	29	38	20
Interval between tests, d (range)	NA	20 (8–61)	53 (36–90)	242 (110–364)	534 (371–945)
HEV RNA positive	151 (100.0)	80 (63.0)	7 (24.1)	0	0
HEV IgM positive	63 (42.0)	95 (74.8)	29 (100.0)	30 (78.9)	12 (60.0)
HEV IgG positive	45 (43.0)	99 (78.0)	28 (96.6)	38 (100.0)	18 (90.0)
Biochemical parameters					
** Bilirubin >1.1 mg/dL**	4 (2.6)	4 (3.1)	1 (3.4)	NA	NA
** AST >80 U/L**	10 (6.6)	6 (4.7)	0	NA	NA
** ALT >80 U/L**	15 (9.9)	12 (10.2)	0	NA	NA
** γ-glutamyltransferase > 55 U/L**	24 (15.9)	18 (15.7)	3 (10.3)	NA	

*Values are no. (%) unless indicated otherwise. ALT, alanine aminotransferase; AST, aspartate aminotransferase; HEV, hepatitis E virus; NA, not applicable.

A physician at the Liver Unit of Vall d’Hebron Hospital reevaluated 29 persons who had detectable HEV RNA or altered bilirubin/transaminase levels at the 1-month follow-up. At this point, all persons had shown seroconversion to HEV IgM (28 also to HEV IgG), 24.1% were still HEV RNA positive, only 1 person had an increased bilirubin level, and all persons had standard levels of transaminases. Despite 2 patients who had mild weakness when raising an arm, an electromyography ruled out proximal myopathy. The remaining persons did not have signs of acute hepatitis E or any extrahepatic manifestation.

In this cohort of HEV RNA‒positive blood donors, the maximum duration of detection of RNA was 71 days after blood donation. Long-term follow-up (6 months to >1 year) showed that none of the HEV RNA‒positive donors showed development of chronic HEV infection, and 79% continued to be HEV IgM positive 6 months after the HEV RNA‒positive donation. All these donors remained HEV IgG positive, and none were reinfected with HEV.

Thirty archived samples obtained during the 6 months before the HEV RNA‒positive donor identification were analyzed by HEV RNA in individual sample (95% limit of detection 11 IU/mL). Twenty-nine samples were HEV RNA negative in individual sample testing, but 1 archived sample was positive. This sample corresponded to a platelet apheresis that had been collected, tested negative in minipools of 16 samples, and transfused. The viral load was ≈40 IU/mL. In this instance, the hospital that had received the HEV RNA‒contaminated platelet apheresis was informed of potential risks and advised to take appropriate action. The physician informed the blood bank center that no signs of acute hepatitis E were observed, and 3 months after the transfusion, the recipient was negative for HEV RNA and HEV IgG/IgM.

### Posttransfusion HEV Infection Surveillance

The Catalonia blood bank provides all blood components (plasma, platelets, and erythrocyte concentrates) to all hospitals in Catalonia. Half of blood component recipients are immunocompromised patients. Because we implemented the HEV RNA universal screening in minipools of 16 samples during November 2017, no new cases of transfusion-transmitted hepatitis E have been reported in Catalonia.

## Discussion

In Spain, universal screening of blood donations for HEV is not mandatory. During 2013, the prevalence of HEV RNA in blood donations in Catalonia was 1 case/3,333 donations, tested individually, whereas HEV IgG seroprevalence was 20% (*16*). Another study conducted in south-central Spain during 2017‒2018 showed a similar prevalence of HEV infection among blood donors (1 case/2,828 donations) (*17*). Furthermore, 2 cases of HEV transfusion-transmitted infection by supernatant of cryoprecipitate and erythrocyte concentrate were documented in Catalonia during 2015 and 2017 (*11*,*12*). The most comprehensive study in transfusion-transmitted HEV infection was conducted in the United Kingdom and showed an HEV transfusion-transmitted rate of 42% (*10*). Taking into account all these results, the blood bank of Catalonia implemented universal HEV RNA screening in pools of 16 samples during November 2017.

HEV RNA screening of 653,800 blood donations during 2017‒2020 showed a high prevalence of HEV infection in Catalonia. We detected 151 HEV RNA‒positive donations, corresponding to a prevalence of 1 case/4,341 donations, which is consistent with the prevalence reported in the same cohort during 2013, although in this study, HEV RNA was detected in individual samples (*16*). This proportion of HEV-infected donors is similar to those reported by Canada (1 case/4,615 donations) and many countries in Europe, including England (1 case/2,848 donations 1 case/4,781 donations), Ireland (1 case/4,745 donations), Austria (1 case/5,369 donations), and Belgium (1 case/5,448 donations) (*13*,*17**–**20*). Although we observed lower prevalence than Germany (1 case/1,241 donations), France (1 case/1,317 donations), and the Netherlands (1 case/1,987 donations) (*21*–*23*), the results cannot be compared directly because different pool size and methods have been used. The United Kingdom, the Netherlands, and Ireland have implemented universal HEV screening, whereas other countries, such as Germany and France, have implemented selective HEV RNA screening for use in high-risk patients. Currently, only 2 of 17 blood regional establishments in Spain have implemented universal HEV RNA screening: the blood bank of Asturias in northern Spain (implemented during 2020) and the blood bank in Catalonia. Our results indicate that Spain should be considered an area of medium to high endemicity for HEV, comparable with the rest of Europe, and universal or selective screening for HEV RNA should be carefully considered.

HEV-infected donors were distributed around Catalonia, and no outbreaks were observed. The highest HEV RNA prevalence among donors in Catalonia was observed in the 56–65 year-old persons (2.85 cases/10,000 donations), in contrast to what was previously reported in England and Ireland, where the highest incidence was seen in young donors (18‒24 years of age) (*13*,*19*).

As in the United Kingdom, HEV RNA‒positive donors in Catalonia are deferred for 6 months (*19*). In the immunocompetent population, the course of HEV is usually asymptomatic, with an estimated manifestation index of 13% (*22*). In our study, 15 of 151 HEV-positive blood donors showed moderate and transient increases of alanine aminotransferase levels, although none had hepatic or extrahepatic manifestations either at the time of donation or at 6-month follow-up. In our study, HEV RNA clearance in infected donors was achieved after a maximum of 71 days, similar to what was observed in another study in Spain (*17*). None of the infected donors showed development of chronic HEV infection, and none were reinfected at follow-up. These observations are in concordance with the clinical guidelines by which spontaneous viral clearance in immunocompetent subjects typically occurs in the first 3 months of infection, and infection persisting beyond this period is recommended to be considered a chronic infection (*24*). A total of 58% of HEV RNA‒positive donors were seronegative at the time of donation, which contributes to transmission because the antibody status of the donor could be a determinant in preventing HEV transfusion transmission (*10*). Finally, HEV IgM was detected 1 year after the HEV exposure in immunocompetent persons, indicating that this detection is not a good clinical biomarker of acute HEV infection and that nucleic acid amplification testing (NAT) should be performed (*25*).

Our study also demonstrates the diversity of HEV strains circulating in Catalonia. On the basis of phylogenetic analysis of 91 HEV isolates, we determined that all infections were caused by HEV genotype 3 and showed the dominance of subgenotype 3f variants (80%). Genotype 3 virus causes zoonotic infections in pigs, wild boars, and deer. It is clear that most HEV infections are acquired in Spain because only 33% of donors reported travel abroad in the 2-month period before donation (*26*).

We identified consumption of undercooked meat or pork-derived foods (liver pate or sausages) in 42% and 66% of HEV-infected donors, respectively. A study analyzed the presence of HEV RNA in different organs and tissues of 45 apparently healthy pigs from 9 slaughterhouses in Spain (*27*). The findings of this study indicate that pig liver pate and sausages could be the route of HEV infection. In addition, 63% of HEV RNA‒positive donors reported seafood consumption during the 2 months before donation. A recent study conducted in Galicia (northwestern Spain) analyzed 168 shellfish samples from this region, and HEV RNA was detected in 41 (24.4%) samples, although all isolates belonged to subgenotype 3e (*28*). In our study, none of HEV-infected donors had subgenotype 3e, suggesting that shellfish consumption was not a probable route for HEV transmission in our population. Finally, half of HEV-infected donors reported consumption of nonbottled water. Although HEV strains belonging to genotype 3 were frequently detected at low concentrations in urban sewage and biosolids or sewage containing swine feces, they were not detected in the river water samples or drinking water treatment plants studied in Spain (*29*,*30*). In addition, 2 persons reported consumption of game meat (wild boar) shortly before donation. Because previous studies detected HEV RNA subgenotypes 3f and 3c in wild boars from Catalonia (*25*), they were the most probable route of HEV acquisition for these 2 cases.

We detected only 1 donation that was missed by initial screening of minipools containing 16 samples. This donation had low viral load (<40 IU/mL) and did not cause a transfusion-transmitted HEV infection in the recipient. A previous study estimated that the infectious dose is ≈20,000 IU of HEV RNA (*10*,*21*). For our case, because the apheresis platelet donation had a volume of 300 mL of plasma, the recipient received 12,000 IU of HEV RNA, which might not be sufficient to induce an HEV infection. As a limitation of this study, although no additional transfusion-transmitted HEV infections have been reported in Catalonia since implementation of HEV NAT in blood donations, it is difficult to quantify the number of infections that have been prevented by NAT screening because HEV infections are often underdiagnosed.

In conclusion, 151 HEV infected donors have been detected during 2.5 years of HEV RNA universal screening strategy in Catalonia (minipools of 16 samples), and no cases of HEV-transfusion-transmitted infection have been reported in our region. Moreover, most HEV-infected donors were asymptomatic, none showed development of chronic HEV infection, and no HEV reinfection was observed. Our data support the suggestion that HEV RNA screening of blood donations provides safer blood for all recipients, especially for immunosuppressed patients.
